# Contribution of Cyanotoxins to the Ecotoxicological Role of Lichens

**DOI:** 10.3390/toxins13050321

**Published:** 2021-04-29

**Authors:** Dobri Ivanov, Galina Yaneva, Irina Potoroko, Diana G. Ivanova

**Affiliations:** 1 Department of Biochemistry, Molecular Medicine and Nutrigenomics, Faculty of Pharmacy, Medical University “Prof. Dr. Paraskev Stoyanov”, 9002 Varna, Bulgaria; galina.yaneva@mu-varna.bg (G.Y.); divanova@mu-varna.bg (D.G.I.); 2 Department of Food and Biotechnologies, School of Medical Biology, South Ural State University, 454080 Chelyabinsk, Russia; potorokoii@susu.ru

**Keywords:** toxic lichens, toxic cyanoprokaryotes, lichen toxins

## Abstract

The fascinating world of lichens draws the attention of the researchers because of the numerous properties of lichens used traditionally and, in modern times, as a raw material for medicines and in the perfumery industry, for food and spices, for fodder, as dyes, and for other various purposes all over the world. However, lichens being widespread symbiotic entities between fungi and photosynthetic partners may acquire toxic features due to either the fungi, algae, or cyano-procaryotes producing toxins. By this way, several common lichens acquire toxic features. In this survey, recent data about the ecology, phytogenetics, and biology of some lichens with respect to the associated toxin-producing cyanoprokaryotes in different habitats around the world are discussed. Special attention is paid to the common toxins, called microcystin and nodularin, produced mainly by the *Nostoc* species. The effective application of a series of modern research methods to approach the issue of lichen toxicity as contributed by the cyanophotobiont partner is emphasized.

## 1. Introduction

Lichens are widely distributed on Earth and their specific features deserve comprehensive research as these contribute to special ecological relationships with other organisms and to numerous applications by humans. Biological activity of lichen secondary substances may exert harmful effects on a variety of microorganisms, animals, and human beings. The toxicity of some lichen species accounts for interdisciplinary studies aiming at revealing the intimate mechanisms of the interactions between these symbiotic entities, on one hand, and natural environment, on the other hand. This review addresses some aspects of lichen toxicity that may be contributed to the cyanoprokaryote photobionts. In the present concise survey recent data about the ecology, phytogenetics, and biology of lichens with respect to the associated toxin-producing cyanoprokaryotes in different habitats around the world as reflected in the recent literature available, have been summarized.

## 2. Some Ecological and Phylogenetic Aspects of Lichen Symbiosis

Lichens are symbiotic entities accommodating a fungus, the mycobiont, and a photosynthetic partner—the photobiont, either algae, or cyanoprokaryotes/cyanobacteria, or both. In most cases, the symbiotic photobiont is a green alga (Chlorophyta), and more than 200 different cyanobacteria or green algae are found to act as photobionts. Approximately 21% of all known fungi are capable of acting as a mycobiont partner. Almost all of the lichen-forming fungi (about 98%) belong to Ascomycetes, and almost half of the Ascomycetes are lichenized [1]. The lichen when perceived as communities may accommodate a diverse array of associated microscopic organisms, including other associated fungi, bacterial communities, and even viruses [2].

Lichens inhabit all terrestrial biomes and diverse ecosystems—from deserts to high elevation mountains and the Arctic tundra. That is how lichens encounter for more than 20,000 species identified so far. Not only climate, but also different substrates, quite often very inhospitable, accommodate lichens and, thus, contribute further to lichen diversity. Lichens are ubiquitous on tree stems and barks, leaves, and soil, which often occupy habitats less favourable from other photosynthetic lifeforms [3]. Giving the fact that about 6–8% of the ground layer on the Earth is inhabited by lichens, their impact on terrestrial communities and ecosystems functioning is very substantial. In addition, they contribute to the ecosystem biogeochemistry [4].

About 13% of all lichens accommodate cyanoprokaryotic partner as a primary or secondary photobiont (bipartite or tripartite symbioses, respectively). Cyanolichens are a polyphyletic group. Their representatives belong to different orders of Ascomycota, most often families of the order Peltigerales, and especially genera *Nephroma* and *Peltigera*, where cyanolichens are mainly bipartite foliose with ubiquitous distribution. The predominant photobionts show a great variety from a phylogenetic point of view. In a study by variation-partitioning analyses, the effect of climate, of specific substrates and habitats, and of mycobionts on the distribution of photobionts has been demonstrated. Lichen lineages at the species level with more symbiotic partners occupy wider climatic niches due to different niches of their partners. The ability of lichens to inhabit substrates that accumulate high amounts of heavy metals is attributed to their interaction with various photobionts tolerant to toxicity [5].

While most lichen individuals contain a single photobiont, which is green algae, there are many symbiotic entities, e.g., within *Peltigera* lichens in which the photobiont partner is a cyanobacteria (bipartite symbioses). In tripartite symbioses, the photobiont in the lichen thallus is a green alga (*Coccomyxa*), and there is a second photobiont associated on the thallus surface, forming cephalodia—cyanobacteria (*Nostoc*) [6].

The evolutionary and ecological adaptation of lichens as successful symbiotic systems is demonstrated by the interactions between the fungal partners from a monophyletic section of a single genus worldwide and the photobiont partners belonging to diverse phylogroupes. Examining 250 lichen specimens of 42 *Peltigera* species of the monophyletic section *Polydactylon* and their interaction with 43 photobiont phylogroups, 91 different interaction pairs were identified. The interactions were highly modular and anti-nested, which suggested strong preferences [7,8]. Exploring the modularity of an evolutionary lichen network of interactions could help in understanding the metabolic relationships between related species, including production of biologically active metabolites and their release into the environment.

Cyanobionts of two coastal species of *Lichina* lichen (*Lichina confinis* and *Lichina pygmaea*) were studied for phylogenetic relationships using 16S rRNA and phycocyanin operon markers. It was observed that niche differentiation, symbiont separation, and the distribution of photobiont lines by ecological and geographical gradients contribute to the patterns of photobionts’ preference. Specimens collected in the North-oriented coasts of Europe use different cyanobiont lineages than those from West-facing Atlantic coasts. Thus, the ecological specialization expressed as cyanobiont haplotypes not shared between *Lichina* species proves that two ecologically differentiated pools exist in the supratidal and intertidal zones [9].

The phylogenetic diversity of the mycobionts and cyanobionts of *Peltigera* species from Southern Chile and Antarctica are assessed [10]. A total of 186 samples are collected and the taxonomic status of each symbiont is identified by analyzing the diversity of DNA coding for the small subunit rRNA of the mycobionts and for the large subunit rRNA of the cyanobionts. DNA was extracted from the lichen thallus and the established sequences allowed to associate all the cyanobionts with the *Nostoc* II clade proposed by O’Brien et al. [11] and with the *Peltigera* guild proposed by Rikkinen et al. [12]. The fact that 77 *Nostoc* sequences that did not exhibit strong geographic patterns observed in previous investigations [13,14] suggests that some *Nostoc* taxa have a wider distribution [10].

More than 150 new lichens have recently been reported from European Paleogene amber [15]. Fossil fungi have recently been used to calibrate evolutionary analyses in studies on the species of Ascomycota, which is the largest group in the fungal kingdom [16,17,18]. Within them, some lichen-forming genera exist, from Paleogene *Anzia* (Parmeliaceae, Lecanoromycetes), *Calicium* (Caliciaceae, Lecanoromycetes), *Chaenotheca* (Coniocybaceae, Coniocybomycetes), and from Miocene *Phyllopsora* (Ramalinaceae). Diversity of ecological preferences within each lineage ensures that, from each group, at least some taxa exists that can survive global crises and recover quickly [15].

## 3. Lichen Toxicity as Contributed by the Cyanophotobiont Partner

Lichen survival under harsh environmental conditions is enabled by the successful symbiotic relationship among the fungal and the photosynthetic partner. Their resistance is mainly due to their ability to survive in a metabolic resting state for long periods of time and to their slow metabolism. Due to lichens’ broad ecological diversity, their metabolites may vary significantly and, accordingly, exert differential toxicity, thus, widening the types of biological effects and representing a broad arsenal of biological weapons that are used for self-defense to repel intruders. Lichens produce many biologically active substances (over 800) [19], comprising many classes of compounds. It should be noted that many of these metabolites are produced by lichens as symbiotic systems only, and not by any of the isolated symbiont partners [20]. For example, even the most studied toxic substance produced by the fungal partner, usnic acid, seems to be an exclusively secondary lichenic compound with one reported exception, which is a product of non-lichenized ascomycetes [21]. In two other cases, it is produced by isolated mycobionts [22]. A single publication describes the presence of a similar usnic acid compound in a fungus with phytopathogenic properties [23].

Furthermore, an observation establishes a correlation between the lichen slow growth in habitats with scarce resources with the presence of higher levels of defense substances [24]. For such long living communities as lichens are, this is a mechanism of an evolutionary adaptation. Taxonomical diversity of lichens and the ecological diversity, along with the difficulty to culture lichens under controlled conditions long enough to mimic environmental impact, makes it difficult to analyse lichen metabolites’ bioactivity and toxicity.

Most studies on the secondary toxic metabolites produced by the lichen photobionts are carried out on free living cyanoprokaryotes and algae in relation to aquatic blooms. The symbiosis between the fungus and the photobiont induces major changes in the metabolism and morphology of lichens. The studies of the production of toxic metabolites by the photobiont in lichen symbiosis are scarce. Typically, the photobiont partner of lichens inhabiting low-resource habitats is an N_2_-fixing cyanobacteria, such as *Gloeocapsa* or *Nostoc* [20]. Cyanoprokaryotes are photosynthetic organisms ubiquitously distributed in a variety of aquatic and terrestrial habitats. They live in fresh, brackish, and sea waters, on soil, on plant leaves, or in different symbioses, including lichens, and even may inhabit unfavourable environments of high salinity or extreme temperatures, such as deserts and hot springs [25]. The ecological impact of these ancient and diverse cyanoprokaryotes in terms of their toxicity is major as they produce harmful algal blooms and release metabolites toxic to other species, threatening the equilibrium of the ecosystems and human health [26].

In recent years, genomic research provides tools for water quality screening and forecasting algae blooms well in advance by identifying and characterizing the specific strains and their toxic products to manage the toxicity risk. A total of 194 cyanobacterial strains from terrestrial habitats and brackish and fresh waters in Brazil, Finland, Czech Republic, Denmark, and Bermuda are screened using a disc diffusion assay [27]. For the first time, two *Anabaena* strains that produce the potent cytotoxin scytophycin are isolated from gastropods living in brackish coastal waters in Finland. Two other strains of the benthic cyanoprokaryotes *Nostoc* and *Scytonema* from the Baltic Sea coast produce scytophycin as well [27]. Scytophycin production is also established in *Nostoc, Scytonema*, *Cylindrospermum,* and *Tolypothrix* strains isolated from terrestrial habitats [28]. Other products, belonging to the group of microcystins, which are very toxic to invertebrates, are isolated from microalgal pelagic and benthic freshwater blooms with the prevalence of *Microcystis aeruginosa* [29]. Along with another cyanobacterial species, *Raphidiopsis raciborskii, Microcystis aeruginosa* occupies and dominates environmental niches that embed numerous highly variable strains, where mutual interactions contribute to potentially toxic blooms and distribution of both species worldwide [30]. The cyclic heptapeptide microcystins are produced by many different cyanobacteria, e.g., *Microcystis, Oscillatoria, Aphanocapsa, Cyanobium, Arthrospira, Limnothrix, Phormidium, Hapalosiphon, Anabaenopsis, Nostoc, Synechocystis*, *Anabaena*, *Hapalosiphon*, *Microcystis*, *Nostoc*, and *Planktothrix* [31]. These are chemically stable molecules and few bacteria in aquatic ecosystems are known to be able to degrade them [32].

Toxin production in coastal marine waters and freshwaters is an increasingly emergent worldwide problem [33]. Of a great interest to scientists and water quality management bodies is the development of accurate tests to directly and simultaneously analyse microalgal toxins in water or the terrestrial samples to assess the risk for an ecosystem. A novel flow cytometric toxicity test for the assessment of the environmental risk in freshwater cyanoprokaryote multispecies systems analyses the contribution of each microalgal species by quantifying the difference in their fluorescence and light scattering properties. The test has been applied for tropical freshwater environments with *Pediastrum duplex*, *Chlorella sp*., *Monoraphidium arcuatum*, and *Nannochloropsis*-like sp. [34]. An ultraperformance liquid chromatography-tandem mass spectrometry (UPLC-MS/MS) method with a solid phase extraction step employing isotopically labelled internal standards identifies 13 different cyanoprokaryote toxins in freshwater sources, microcystins, and nodularin among them [35]. By using a time-efficient liquid chromatography mass spectrometry (LC-MS) precursor ion screening method and an ADDA-ELISA (enzyme-linked immunosorbent assay kit for microcystins), six different microcystins are detected in eight out of 26 samples from the Arctic, in accordance to other reports about similar polar habitats [36]. By applying a variety of molecular methods targeting 16S rRNAs and genes related to the production of microcystins, *Nostoc* sp. are established as putative toxin producers.

The cyanobacterial toxins, microcystin and nodularin, previously primarily associated with harmful water blooms, are typical for terrestrial environments also, where they live in symbioses with the mycobiont in different lichen species [25]. To summarize, a number of cyanoprokaryotic partners in the lichen symbiotic entities produce secondary toxic metabolites, known as microcystins and nodularins, as being the most widely represented ones produced mainly by the *Nostoc* species. With regard to the findings related to *Nostoc* toxicity, a hypothesis about the macroevolution of lichens envisions a correlation between the specificity of the mycobiont and the inheritance of a specific pool of *Nostoc* phylogroups with occasional shifts between cyanobiont pools [8]. In this respect, a better understanding of the relative contribution of the evolutionary vs. ecological drives for the unraveling and deciphering of the current status and role of lichens is necessary [7].

## 4. Contribution of *Nostoc* Species to Toxin Production in Lichens

High concentrations of the very toxic microcystins and their variants are established in different *Nostoc* strains. The total microcystin concentration in the terrestrial cyanobacterium strain IO-102-I is 0.2 μg/mg (dry weight) [37]. This *Nostoc* IO-102-I strain isolated from a lichen association is the third *Nostoc* and the second terrestrial strain that is known to produce six different microcystins, one of the most notable of which, due to its high hepatotoxicity, is the rare microcystin-LR. Scattered terrestrial *Nostoc* distribution could be the reason for the rarely established microcystin production in the terrestrial environment [37]. Strains of the genus *Nostoc* isolated from an aquatic environment are also found to synthesize microcystins: *Nostoc* sp. strain 152 from Lake Sääskjärvi in Finland [38] and *Nostoc* sp. strain DUN901 isolated from brackish water of Barrow Ski Club Lake in the United Kingdom [39], where microcystin toxicity expands to inhibition of the green algae photosynthesis [40]. Gene analysis of 803 lichen thalli representing 23 different cyanolichen genera from different parts of the world identifies the presence of the microcystin coding gene, the *mcyE* gene, in 98 cyanolichen specimens [25], and the toxin presence is confirmed in 42 of these lichens. A second type of toxin, nodularin, is established in three of the specimens. It is worth noting that toxin-containing lichen thalli are found in all the geographical regions with the highest percentages of thalli containing toxins and/or the *mcyE* gene recorded for Scotland (58%), Norway (30%), and Oregon, USA (21%). There are 52 different microcystin variants from which 20 variants have a relative intensity over 10% at least in one lichen sample [25].

The cytotoxicity of 82 *Nostoc* strains isolated from different terrestrial habitats varies between different climate regimes [41]. The overall occurrence of cytotoxicity is 33% and differs significantly among strains, isolated from sites with a different microclimate in support of the hypothesis that cytotoxicity depends on the environmental factors. Thus, the proportion of high cytotoxic strains against less toxic strains increases for isolates from tropical habitats (9%) to deserts (14%), cold regions (36%), isolates from sites with a continental climate (45%), and was highest among symbiotic strains (60%). Interestingly and in support of the above hypothesis, different metabolites were the causative reason for the particular toxic effect of strains isolated from a specific microsite [41]. The amount of microcystins synthesized also varies depending on environmental factors, such as light, temperature, or physiological stress [42], under which *Nostoc* sp. strain 152 produces the highest amounts of the toxic microcystin heptapeptide.

*Nostoc* species are also recognized to produce the cyclic pentapeptide hepatotoxic nodularins, and, especially, nodularin-R, which is a widespread cyanotoxin in aquatic blooms, a protein phosphatase inhibitor, and a tumour promoter. A study on the benthic *Nostoc* sp. CENA543 from saline-alkaline lake in Brazil associates the free-living *Nostoc* with nodularin production at levels comparable to those of the toxic bloom dominating in aquatic ecosystems *Nodularia spumigen* [43].

The first direct evidence that nodularin may be produced by other than *Nodularia* cyanoprokaryote genus comes from the finding that cycad and lichen symbiotic *Nostoc* strains also synthesize it [25,44].

Symbiotic *Nostoc* strains are typically found in lichens from the genuses *Peltigera* and *Nephroma* (Peltigerales, Ascomycota), which often inhabit regions with extreme temperatures. Virtually all *Collema*, *Leptogium*, *Nephroma*, *Peltigera*, *Pseudocyphellaria*, and *Sticta* species associate with *Nostoc* [45]. *Nostoc* is the most common photosymbiont in lichens and 13% of all lichen species have cyanoprokaryote symbiont partners [12]. Symbiotic *Nostoc* strains are phylogenetically grouped into two distinct lineages, which associate with different lichen-forming fungi. The first group of *Nostoc* strains are those within the bipartite species of *Nephroma*, *Sticta*, and *Pseudocyphellaria* and the other group is involved within the Peltigeralean lichen species [46].

A significant part of species within the Nostocales produce structurally diverse biologically active strain-specific secondary metabolites, mainly polyketides and non-ribosomal peptides. A number of them also exert different types and levels of toxicity and the cyclic pentapeptide nodularins and cyclic heptapeptide microcystins are the most widespread cyanotoxins in freshwater and brackish toxic blooms in various climatic regimes worldwide [47,48]. Often an individual strain is characterized by the ability to produce several classes of metabolically active compounds [49]. It is a challenge to isolate the lichen photobiont and study the cell culture for cyanotoxicity in vitro. A study has established a lichen-associated *Nostoc* strain from the bipartite *Protopannaria pezizoides* to produce microcystins under such experimental conditions [37]. Another study demonstrated the in-situ production of the hepatotoxic microcystins by cyanobacterial symbionts of the tripartite cyanolichen *Peltigera leucophlebia* [31], both from cephalodia and from a symbiotic *Nostoc* strain. Only one of the several genetically identical symbiotic *Nostoc* strains present in the lichen was found to produce the toxin in culture. Three microcystin variants were produced by the *P. leucophlebia* cephalodia.

The microcystin structure is so variable from one strain to another that more than 240 variants of microcystins have been established so far in contrast to the modest structure variability of nodularins (about ten different types totally) [33]. Toxicity type of the secondary metabolites produced by the Nostocales species also varies from hepatotoxicity of microcystins to cytotoxicity of cylindrospermopsin and neurotoxicity of anatoxins and saxitoxins, as reviewed by Cirés and Ballot (2016) with regard to the phylogeny and ecology of bloom-forming cyanoprokaryotes [50]. These variations may be due to the ecological environment or experimental conditions, as in the cell culture experiments. Thus, high deviations in humidity and temperature are related to increased toxin production in lichens from Oregon (USA), Argentina, Scotland, and Norway, and a greater number of nodularin variants are established in lichen specimens from Argentina and Kenya [25].

## 5. Distribution of Toxin-Producing Cyanobacteria in Lichen Species

There is a great variety of symbiotic cyanoprocaryotes, which may be presumably divided into small, vertically transmitted populations that undergo a similar evolution, i.e., are more sensitive to random events, increasing their genetic diversity. Cyanolichens associated with toxin-producing photobionts occupy a special place, further providing an important environment for the diversification of cyanosymbionts [25].

The cyanotoxin-producing photobionts are unevenly and rather sporadically distributed among the cyanolichens, specifically *Nostoc* among Peltigeralean lichens as well as among abundant, common, and widespread macrolichens *Leptogium, Lobaria*, *Nephroma*, *Peltigera*, *Pseudocyphellaria*, and *Sticta.* This suggests that the production of toxins is hardly the primary reason for *Nostoc* to choose a fungal host and the fungus- cyanoprocaryote match is a more complex phenomenon.

High-performance liquid chromatography mass spectrometry (HPLC-MS) detected microcystin production in lichen-associated cyanobacteria is found in a pooled cephalodia sample from the tripartite *Peltigera leucophlebia* thallus [31]. The symbiotic *Nostoc* strain from the same lichen specimen is cultured and sequencing analysis confirms the presence of *mcyE* gene coding for the microcystin synthetase. Only one of all *Nostoc* 16S rRNA haplotypes present in the lichen sample is established in the toxin-producing cultures, while all nine cephalodia isolated and cultured strains produce the same 19 microcystin variants.

Direct detection of microcystins and genetic screening of 803 lichen specimens from all over the world for the presence of the cluster encoding microcystin synthetase responsible for the microcystin production identifies one or the other in 12% of the analyzed lichen specimens [25]. Furthermore, more than 50 different microcystin variants are found, with many of them rarely reported from non-lichenised cyanobacteria. In addition, nodularin presence in significant amounts is reported for some lichen thalli specimens.

In 94 lichen specimens with *mcyE*-gene containing cyanobacteria, 41 different 16S rRNA genotypes are identified. Only one 16S rRNA genotype was identified from every sample from which an *mcyE* gene sequence was amplified [25]. Some monophyletic genera like *Planktothrix*, *Microcystis*, *Anabaena*, and *Nodularia* are identified by this 16S rRNA lineage. The sequences of lichen-associated *Nostoc* show a close relationship with the aquatic *Nostoc* sp. strain 152. *Nostoc* in lichen symbiosis forms several lineages including associations with host fungi that have wide distribution like *Nephroma* guild (A–F) and the *Peltigera* guild (G–L) [51], thus, indicating that the lichen specimen’s geographical origin does not predict which *Nostoc* symbiont will associate with the fungi. In addition, the production of toxins in detectable amounts is not limited to well-defined *Nostoc* groups [25].

A wide-scale analysis of ecological and geographical distribution of different cyano groups included 803 Peltigeralian lichen specimens from the entire world [52]. Microcystin and nodularin toxins were established in specimens from *Lobaria*, *Nephroma*, *Peltigera*, and *Sticta* species. Mainly lichens from Peltigeraceae, such as *P. membranacea* and *P. degenii*, and from Nephromataceae (*N. parile* and *N. cellulosum*) were presented by toxin-producing cyanobionts—11% of all *Peltigera* specimens contained a toxin-producing cyanopartner. The *Nostoc* diversity patterns correlated with the microcystin variants and similar *mcyE* genotypes represented the lateral gene transfer between symbiotic cyanobacteria groups [52].

In addition, many secondary toxic substances with a potential defensive role against herbivores are produced by the symbiotic mycobiont and, together with the cyanopartners, contribute to this chemical defense [25]. The interrelationships between these biologically active chemical compounds of fungal and cyanoprokaryote origin have not been investigated yet due to both the wide molecular diversity and the multiplicity of symbiotic partnerships.

## 6. Conclusions

Substantial advances in international lichen toxicity research have been recently achieved. Additional interdisciplinary efforts are needed to elucidate the still insufficiently revealed intimate mechanisms of lichens’ toxic actions in maintaining the ecological diversity and balance under the conditions of unfavourable world environmental changes.

## Data Availability

Data sharing not applicable.
